# Survival and Risk Factors Among Dialytic Acute Kidney Injury Patients After Cardiovascular Surgery

**DOI:** 10.21470/1678-9741-2017-0184

**Published:** 2018

**Authors:** Andrea B. V. Silva, Agueda Maria Ruiz Zimmer Cavalcante, Fabio P. Taniguchi

**Affiliations:** 1 Instituto Dante Pazzanese de Cardiologia, São Paulo, SP, Brazil.; 2 IAMSPE Post Graduate Health Sciences Program, São Paulo, SP, Brazil.

**Keywords:** Renal Insufficiency, Survival Analysis, Cardiovascular Surgical Procedures, Renal Dialysis.

## Abstract

**Objective:**

Acute kidney injury (AKI) is a frequent postoperative complication after cardiovascular surgery. It has been described as a predictor of decreased survival rates, but how dialysis decreases survival when initiated on the postoperative period has yet to be determined. To analyze the survival of patients who presented postoperative AKI requiring dialysis up to 30 days after cardiovascular surgery and its risk factors is the aim of this study.

**Methods:**

Of the 5,189 cardiovascular surgeries performed in a 4-year period, 157 patients developed AKI requiring dialysis in the postoperative period. The Kaplan-Meier survival curve and log-rank test were used in the statistical analysis to compare the curves of categorical variables. *P*-value< 0.05 was considered significant.

**Results:**

Patient average survival was 546 days and mortality was 70.7%. The need for dialysis on the postoperative period decreased late survival. Risk factors for decreased survival included age (*P*<0.001) and postoperative complications (*P*<0.0003).

**Conclusion:**

The average survival was approximately one year among dialytic patients. Age and postoperative complications were risk factors that determined decreased survival.

**Table:** 

Abbreviations, acronyms & symbols	
AKI	= Acute kidney injury
AMI	= Acute myocardial infarction
BMI	= Body mass index
CABG	= Coronary artery bypass graft
CPB	= Cardiopulmonary bypass
EGFR	= Estimated glomerular filtration rate
EuroSCORE II	= European System for Cardiac Operative Risk Evaluation
ICU	= Intensive care unit

## INTRODUCTION

Acute kidney injury (AKI) is a disease of complex etiology, not fully understood, that often takes place in the postoperative period of cardiovascular surgery^[^^1^^]^. The incidence of postoperative AKI in cardiovascular surgery remains high, approximately 20% of such surgical patients are affected^[^^2^^,^^3^^]^.

In addition to being associated with postoperative complications, longer hospitalizations, risk of infection and greater costs to the health system, AKI is an independent predictor of intra-hospital mortality; since it increases mortality by up to eight times, consequently decreasing the survival of patients^[^^3^^,^^4^^]^.

Among those undergoing cardiovascular surgery, 1% to 5% may require renal replacement therapy^[^^4^^]^. Clinical variables influence the occurrence of postoperative AKI, among other complications, and decreased survival rates^[^^1^^,^^5^^,^^6^^]^. Long-term survival among patients who underwent cardiovascular surgery and acquired postoperative AKI requiring dialysis should be evaluated in a diversified context, since it may confirm information regarding length of survival and its associated risk factors. The aim of this study is to analyze the risk factors in patients who acquired postoperative AKI requiring dialysis after cardiovascular surgery and analyze its survival rates.

## METHODS

This retrospective study was conducted in a large public Brazilian hospital specialized in cardiology. Consecutive patients referred to cardiovascular surgery in a 4-year period were analyzed. Patients older than 18 years old, who underwent cardiovascular surgery and presented AKI requiring dialysis up to 30 days in the postoperative period, were eligible. Those who underwent congenital surgeries and cardiac transplantation were excluded.

Data concerning clinical characteristics were analyzed and assigned into three different groups. Preoperative variables were: sex, age, body mass index (BMI), ethnicity, EuroSCORE (European System for Cardiac Operative Risk Evaluation) II, estimated glomerular filtration rate (EGFR) and comorbidities. Intraoperative variables were: type of surgery, cardiopulmonary bypass (CPB) time, use of vasoactive drugs and the use of an intra-aortic balloon pump. Postoperative variables included: length of mechanical ventilation, the use of vasoactive drugs, red blood cells transfusions, clinical complications, length of stay in the intensive care unit (ICU) and death.

Each patient's BMI was classified according to the guidelines established by the World Health Organization and were used to assess nutritional status^[^^7^^]^. The official website of the EuroSCORE II was used to calculate the patients' score (www.euroscore.org). The Cockcroft-Gault formula was used to estimate the glomerular filtration rate (acute kidney injury stage 1 - increase in serum creatinine of ≥ 0.3 mg/dL or an increase of 50 - 200% from baseline; stage 2 - increase in serum creatinine of 200 - 300%; and stage 3 - increase in serum creatinine of > 300% or serum creatinine level > 4 mg/dL). The last laboratory result concerning the preoperative serum creatinine levels was used and patients were classified according to the classification established by the National Kidney Foundation (2005)^[^^8^^]^. Survival was verified by consulting their ambulatory follow-up records. When necessary, phone calls were performed.

IBM-SPSS (Statistical Package for the Social Sciences) version 19.0 (SPSS Institute, Chicago, Illinois, USA) was used for the statistical analysis. Fisher's exact test was used to compare the categorical variables. The survival of patients was estimated and graphically represented using the Kaplan-Meier curves. The log-rank test was used to compare survival curves. Pairwise comparison was performed when there were more than two groups with Bonferroni correction. Variables that were statistically significant (*P*<0.1) were submitted to multiple analyses according to the Cox's proportional hazards model. Subsequently, the StepWise Backward method was performed to obtain the final model. The variables that presented *P*<0.05 on the latter were considered significant.

This study was approved by the Institutional Review Board at the hospital where the data were collected (protocol No. 4205/12).

## RESULTS

A total of 5,189 cardiovascular surgeries were conducted on adult patients on the period of the study and 157 patients were identified in accordance with the eligibility criteria. The patients' characteristics are presented in Table 1.

**Table 1:** Clinical characterization and pre, intra and postoperative variables of patients who presented postoperative acute kidney injury after cardiovascular surgery and required dialysis. São Paulo, Brazil 2014.

		Characteristics of patients	N	%	Mean/SD	Min/Max
Preoperative variables	Sex	Male	92	59		
		Female	65	41		
	Age (years)	Older than 60 years of age	86	54.8		
		Younger than 60 years of age	71	45.2		
	BMI	Below normal (<18.5)	7	4.7		
		Normal (18.5-24.9)	49	32.9		
		Overweight (25.0-29.9)	49	32.9		
		Obese class I (30.0-34.9)	30	20.1		
		Obese class II (35.0-39.9)	10	6.7		
		Obese class III (≥ 40.0)	4	2.7		
	Ethnicity	Caucasian	123	78.34		
		Mixed	18	11.46		
		Afro-descendent	13	8.28		
		Asian	3	1.91		
	Comorbidities	Hypertension	133	85		
		Dyslipidemia	97	62		
		Diabetes mellitus	63	40		
		Smoking	46	30		
		Prior cardiovascular surgery	41	27		
		Chronic atrial fibrillation	37	24		
		Acute myocardial infarction	28	15		
		Stroke	19	11		
		Chronic obstructive pulmonary disease	9	6		
		Peripheral vascular disease	2	1		
		EuroSCORE II	Median: 3.8% Mean: 6.62% SD: 7.97%		Quartile range 2.08% - 7.37%	Min: 0.75% Max: 52.51%
	Renal Disease Classification	GFR I - Normal	17	11		
		GFR II - Mild	46	29		
		GFR III - Moderate	51	33		
		GFR IV - Severe	8	5		
		GFR V - Renal failure	35	22		
Intraoperative variables	Type of Surgery	VR	78	49.7		
		Myocardial revascularization	56	35.7		
		Combined	20	12.7		
		Thoracic aortic aneurysm repair	3	1.9		
	CPB time	<120 min	82	52.2	Mean: 121.45 SD: 58.13	
		≥120 min	75	47.8		
		Vasoactive drugs	135	86.0		
		Intra-aortic balloon	7	4.5		
	Complications	Infections	113	72.0		
		Atrial fibrillation	71	45.2		
		Bronchopneumonia	69	43.9		
		Cardiorespiratory arrest	33	21.0		
		Surgical wound infection	30	19.1		
		Neurological	19	11.0		
		Low cardiac output	18	11.5		
		Reoperation (hemostasis review)	17	10.8		
		Gastrointestinal (upper GI bleed)	16	10.2		
		Others	34	21.7		
		Transfusion of packed red blood cells	83	52.9		
		Vasoactive drugs	151	96.2		
	Length of ICU stay (days)	1-3	36	23.4		
		4-10	38	24.7		
		11-30	50	32.5		
		>30	30	19.5		
		Time of mechanical ventilation (days)			Mean: 8.3 SD: 15.2	Min:0 Max: 121.0

AMI=acute myocardial infarction; BMI=body mass index; CPB=cardiopulmonary bypass; GFR=glomerular filtration rate; SD=standard deviation; Min=minimum; Max=Maximum; VR=valve replacement

Table 1 shows a predominance of male patients (59%), aged over 60 years (54.8%), Caucasians (78.34%), and overweight (mean BMI of 27.81 kg/m^2^). Comorbidities included: hypertension (85%), dyslipidemia (62%), diabetes mellitus (40%), smoking (30%), previous cardiac surgery (27%). The mean obtained for the EuroSCORE II was 6.62%.

In regard to the classification of kidney disease in the preoperative, 33% presented GFR III (moderate), 29% GFR II (mild) and 22% GFR V (renal failure). In regard to the type of surgery, valve surgeries (49.7%) predominated, followed by myocardial revascularization (35.7%). The average duration of extracorporeal circulation was 121.45 minutes.

Complications identified in the postoperative period were: infections (72%), atrial fibrillation (45.2%), cardiorespiratory arrest (21%), and other less frequent complications (21.7%) that included acute myocardial infarction (AMI), pleural effusion, cardiac arrhythmias, acute arterial occlusion, mesenteric ischemia and liver failure. Survival was estimated at five different points in time over the follow-up period, considering the variables in the pre, intra and postoperative groups, shown in Table 2.

**Table 2:** Association between the pre, intra and postoperative and survival at different points in time with survival and CI 95% (lower lim - upper lim). São Paulo, Brazil 2014.

	Time since surgery	30 days	180 days	1 year	2 years	3 and 4 years	*P*-value^a^
	Overall survival	62.8% (55.3-70.4)	36.2% (28.7-43.8)	32.1% (24.7-39.5)	29.9% (22.6-37.2)	27.6% (20.2-35.0)	
**Preoperative**							
Age	<60 years old	78.6% (69.0-88.2)	46.5% (34.7-58.3)	43.2% (31.4-55.0)	41.5% (29.8-53.3)	38.8% (26.6-51.0)	0.001
	>60 years old	50.0% (39.4-60.6)	27.9% (18.4-37.4)	23.3% (14.3-32.2)	20.9% (12.3-29.5)	19.0% (10.4-27.6)	
Sex	Female	67.7% (56.3-79.1)	30.7% (19.5-41.9)	29% (17.9-40.1)	27.3% (16.3-38.2)	27.3% (16.3-38.2)	0.933
	Male	59.4% (49.3-69.5)	40.3% (30.2-50.5)	34.4% (24.5-44.3)	31.7% (21.9-41.5)	27.6% (17.5-37.7)	
Comorbidities	Diabetes mellitus	72.8% (61.8-83.9)	38.8% (26.6-50.9)	28.2% (16.8-39.6)	28.2% (16.8-39.6)	25.1% (13.4-36.7)	0.642
	Hypertension	64.4% (56.3-72.6)	39.1% (30.7-47.4)	34.1% (25.9-42.3)	31.5% (23.4-39.6)	28.5% (20.2-36.8)	0.26
	Dyslipidemia	65.7% (56.2-75.2)	40.1% (30.3-50.0)	34.4% (24.7-44.1)	31.9% (22.3-41.5)	27.8% (17.8-37.7)	0.373
	Prior AMI	73.4% (55.2-91.7)	45.4% (24.4-66.4)	40.4% (19.5-61.3)	35.3% (14.9-55.8)	23.6% (0.3-46.8)	0.477
BMI	<25	65.5% (52.9-78.0)	40.0% (27.1-52.9)	35.8% (23.0-48.6)	33.4% (20.6-46.2)	30.6% (17.8-43.5)	0.834
	25-29.9	64.7% (51.1-78.2)	34.4% (20.7-48.0)	32.1% (18.6-45.5)	29.8% (16.6-43.0)	29.8% (16.6-43.0)	
	≥30	62.2% (48.1-76.4)	35.6% (21.6-49.5)	28.9% (15.6-42.1)	26.7% (13.7-39.6)	21.3% (7.4-35.3)	
GFR	GFR I – Normal	63.7% (40.4-87.0)	25.5% (4.0-47.0)	19.1% (0.0-38.5)	19.1% (0.0-38.5)	19.1% (0.0-38.5)	0.017
	GFR II - Mild	53.3% (38.8-67.9)	31.1% (17.6-44.6)	28.7% (15.4-42.0)	26.3% (13.4-39.3)	26.3% (13.4-39.3)	
	GFR III - Moderate	60.0% (46.4-73.6)	34.0% (20.9-47.1)	28.0% (15.6-40.4)	25.8% (13.7-38.0)	23.0% (10.9-35.0)	
	GFR IV – Severe^b^	50.0% (15.4-84.6)	12.5% (0.0-35.4)	12.5% (0.0-35.4)	12.5% (0.0-35.4)	12.5% (0.0-35.4)	
	GFR V - Kidney failure^b^	81.8% (68.7-95.0)	59.8% (42.8-76.7)	56.1% (38.7-73.4)	52.3% (34.6-70.0)	46.5% (27.4-65.6)	
Intraoperative							
Type of surgery	Myocardial revascularization	71.4% (59.6-83.3)	44.6% (31.5-57.6)	35.3% (22.7-47.9)	33.4% (21.0-45.9)	27.0% (14.1-39.9)	0.171
	Valve	57.2% (46.2-68.3)	34.6% (23.8-45.3)	33.1% (22.4-43.7)	31.6% (21.0-42.1)	31.6% (21.0-42.1)	
	Correction aorta aneurysm	100.0% (29.0-100)	66.7% (13.3-100.0)	66.7% (13.3-100.0)	33.3% (0-86.7)	33.3% (0-86.7)	
	Combined	55.0% (33.2-76.8)	15.0% (0.0-30.6)	15.0% (0.0-30.6)	15.0% (0.0-30.6)	15.0% (0.0-30.6)	
CPB time	< 120min	63.4% (53.0-73.8)	46.3% (35.3-57.1)	41.1% (30.4-51.8)	37.0% (26.3-47.6)	34.9% (24.1-45.7)	0.054
	≥ 120min	62.1% (51.0-73.2)	24.7% (14.8-34.7)	21.8% (12.2-31.4)	21.8% (12.2-31.4)	19.4% (9.8-29.0)	
	Vasoactive drugs	62.7% (54.5-70.9)	34.7% (26.6-42.8)	31.4% (23.5-39.4)	29.8% (21.9-37.6)	26.8% (18.8-34.9)	0.519
Postoperative							
	Vasoactive drugs	63.4% (55.6-71.1)	35.7% (28.0-43.4)	31.3% (23.8-38.8)	29.0% (21.6-36.4)	26.6% (19.2-34.1)	0.25
	Blood transfusion	57.6% (47.0-68.3)	30.6% (20.7-40.6)	29.4% (19.5-39.3)	25.6% (16.0-35.1)	23.8% (14.4-33.3)	0.16
	Low cardiac output	61.1% (38.6-83.6)	24.4% (3.8-45.1)	24.4% (3.8-45.1)	24.4% (3.8-45.1)	24.4% (3.8-45.1)	0.53
No. of complications	0	88.5% (73.6-100.0)	82.6% (64.8-100.0)	62.4% (38.3-86.5)	62.4% (38.3-86.5)	41.6% (4.6-78.6)	0.0003
	1	71.4% (56.5-86.4)	51.4% (34.9-68.0)	48.2% (31.5-64.9)	41.8% (25.1-58.5)	41.8% (25.1-58.5)	
	2 or more	55.6% (46.0-65.2)	23.4% (15.2-31.6)	21.5% (13.5-29.4)	20.5% (12.7-28.3)	15.3% (6.2-24.4)	
Length of stay in ICU (Days)	1-3	66.7% (51.3-82.1)	44.4% (28.2-60.7)	41.0% (24.7-57.3)	34.2% (18.1-50.3)	29.3% (12.9-45.7)	
	4-10	63.2% (47.8-78.5)	44.7% (28.9-60.5)	36.8% (21.5-52.2)	36.8% (21.5-52.2)	36.8% (21.5-52.2)	
	11-30	34.0% (20.5-47.4)	21.2% (9.6-32.9)	21.2% (9.6-32.9)	21.2% (9.6-32.9)	21.2% (9.6-32.9)	0.088
	>30	100.0% (88.4-100.0)	33.0% (16.0-50.0)	29.3% (12.8-45.9)	25.1% (9.1-41.2)	20.1% (4.5-35.7)	

AMI=acute myocardial infarction; BMI=body mass index; GFR=glomerular filtration rate

a Long-rank test;

b *P*<0.05 pairwise with Bonferroni correct. Value are n (%)

Table 2 shows associations between the pre, intra and postoperative variables and survival rates at different points in time. Overall survival for the first 30 days postoperative was 62.8%. Association between survival and preoperative variables revealed age with *P*-value=0.001; survival rate was lower for patients older than 60 years of age and remained low over time among the patients in this age range.

GFR presented *P*-value=0.017, while patients with GFR V (kidney failure) presented better survival results (81.8%) within 30 days and over time, when compared to the survival of the remaining patients at different stages of kidney disease (GFR I, GFR II, GFR III, GFR IV).

CPB duration was not statistically significant for survival, while the presence of complications was significant (*P*-value=0.0003) in the association between survival and postoperative variables.

Cox regression was performed based on the multivariate analysis and the effect of the covariates age, GFR, CPB time, postoperative complications, length of stay in ICU and survival time was estimated. Data are presented in Table 3.

**Table 3:** Analysis of survival among the variables age, GFR, CPB time, complications and length of stay in ICU (Days). São Paulo, Brazil 2014.

Variables	HR	CI (95%) of HR		*P*-value
		Lower Lim	Upper Lim	
Age	1.039	1.019	1.059	0.0001
GFR I	1.000			
GFR II	0.879	0.421	1.835	0.7323
GFR III	0.851	0.393	1.839	0.6817
GFR IV	1.163	0.404	3.344	0.7785
GFR V	0.614	0.252	1.496	0.2833
CPB time	1.418	0.924	2.175	0.1095
Complications	2.076	1.189	3.623	0.0101
Length of stay in ICU 1-3	1.000			
Length of stay in ICU 4-10	0.736	0.400	1.356	0.3270
Length of stay in ICU 11-30	0.871	0.492	1.540	0.6356
Length of stay in ICU >30	0.381	0.199	0.733	0.0038

CPB=cardiopulmonary bypass; GFR=glomerular filtration rate; HR=hazard ratio; ICU=intensive care unit

The analysis of significant variables regarding survival showed that the older the patient, the greater the risk of death; the risk increased 3.8% per year of life. For CPB time >120 min the risk of death increased approximately 1.6 times when compared to patients with CPB time < 120 min. For patients who presented two or more complications, the risk of death increased approximately 2.3 times when compared to patients with only one complication or no complications. For patients who presented length of stay in ICU > 30 days, decreased 57% the risk of death when compared to patients with length of stay in ICU < 3 days. Data are presented in Table 4.

**Table 4:** Analysis of survival after stepwise backward: variables age, CPB time, complications and length of stay in ICU. São Paulo, Brazil 2014.

Variables	HR	CI (95%) of HR		*P*-value
		Lower Lim	Upper Lim	
Age	1.037	1.019	1.056	0.0000
CPB time	1.600	1.055	2.426	0.0268
Complications (>2)	2.300	1.399	3.780	0.0010
Length of stay in ICU 1-3	1.000			
Length of stay in ICU 4-10	0.776	0.435	1.383	0.3909
Length of stay in ICU 11-30	0.879	0.502	1.540	0.6536
Length of stay in ICU 11-30	0.426	0.227	0.8011	0.0081

CPB=cardiopulmonary bypass; HR=hazard ratio; ICU=intensive care unit

**Fig. 1:**
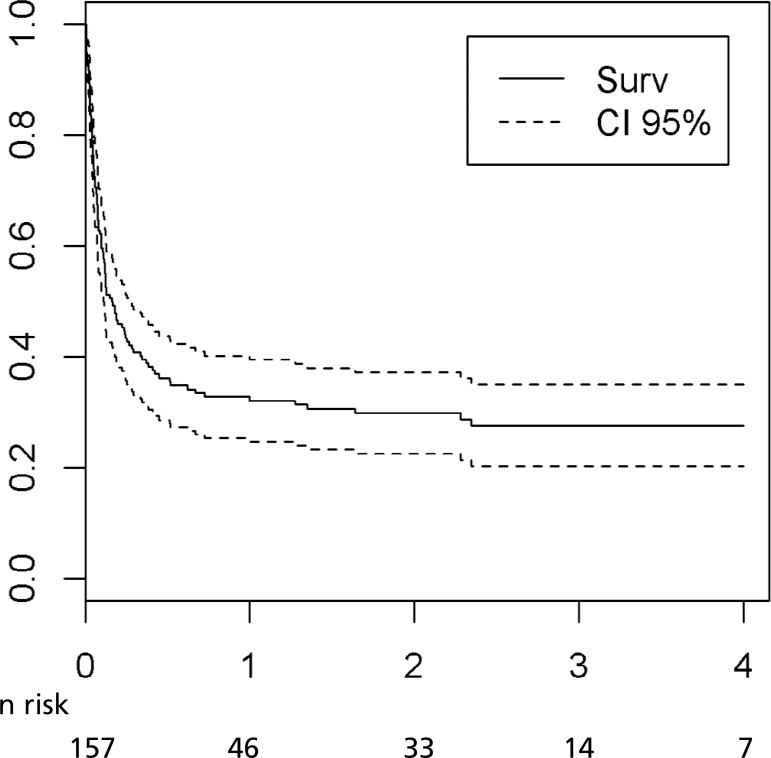
Study patients' overall survival.

Figure 1 represents the patients' overall survival curve. The average time of survival in this study population was 1.5 years, approximately 546 days.

**Fig. 2:**
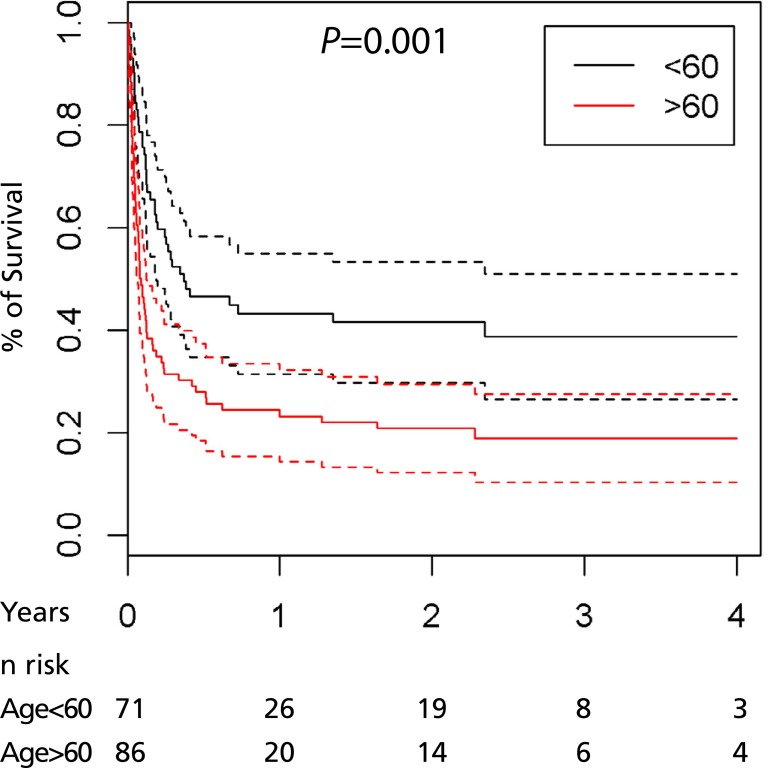
Relationship between the survival and age of patients.

**Fig. 3:**
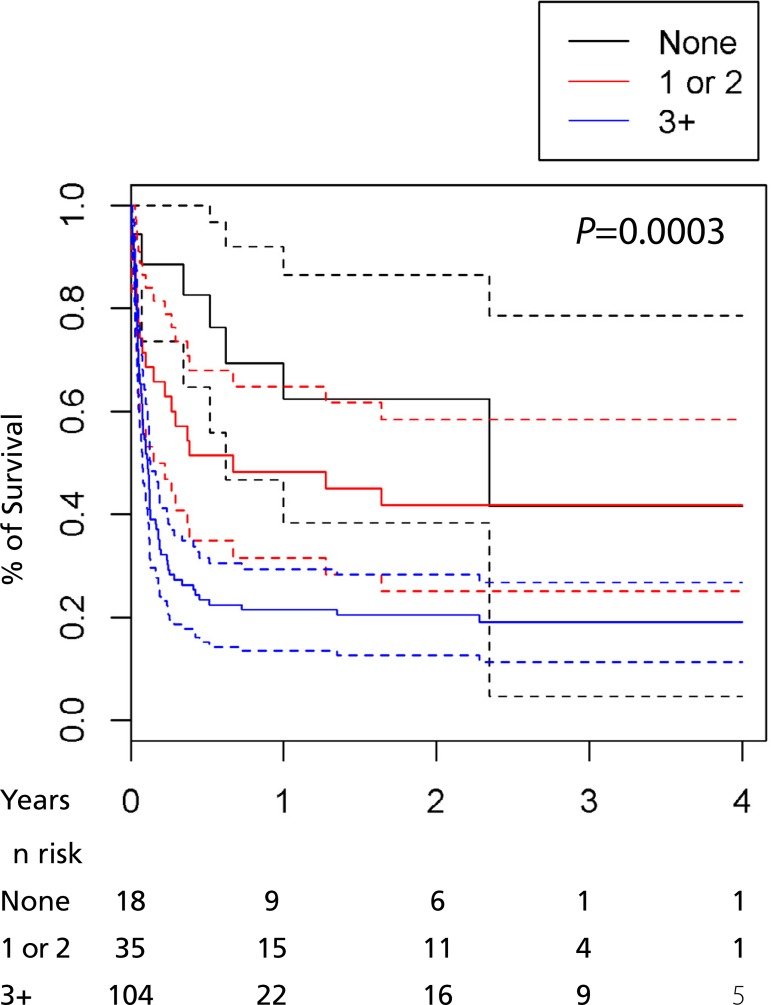
Relationship between the survival of patients and number of complications.

Figures 2 and 3 show survival in regard to the variables age and complications. The dashed line in the first graphic represents only the survival of patients more than the 60 years of age. The first and second dashed lines in the second graphic represent the group with only one complication and two or more complications, respectively.

## DISCUSSION

AKI is a complication that frequently affects patients after cardiovascular surgery and is associated with increased mortality^[^^9^^]^. The aim of this study is to analyze the risk factors in patients who acquired postoperative AKI requiring dialysis after cardiovascular surgery and analyze its survival rates.

The incidence and adverse outcomes associated with AKI in a population who underwent cardiovascular surgery (myocardial revascularization and valve surgery) from 1999 to 2008 reports that the rates of AKI and AKI requiring dialysis after cardiovascular surgery with extracorporeal circulation relates to age, gender, although controversial^[^^10^^]^; type of surgery, heart failure, diabetes mellitus, hypertension, lung disease, peripheral vascular disease, cerebrovascular disease, obesity, sepsis, CPB time, and mechanical ventilation^[^^11^^]^.

Advanced age, as observed in this study, has been related to dialytic kidney failure since the kidneys are prone to aging process^[^^12^^]^. One analysis with 145,911 patients^[^^13^^]^ aged 65 years old or older who had their aortic valve replaced or repaired concomitantly with a myocardial revascularization, between 1991 and 2007, reports an average survival of 13 years for patients aged between 65 and 69 years old, 9 years for those between 70 and 79 and 6 years for patients aged 80 years old or older. Although the type of surgery appears as an important factor associated with decreased survival, it was not significant in this study.

Interestingly, we did not notice in this series relation between BMI and EuroSCORE II to decreased survival since they are well established to dialytic AKI in cardiovascular patients^[^^14^^-^^17^^]^. Other factors predicting AKI were identified in patients who had undergone cardiovascular surgery and developed AKI that required dialysis, such as having a preoperative severe status and preexisting kidney disease^[^^18^^]^.

Despite the reported survival rate in the first 30 days after surgery, it gradually decreased in subsequent months. Similar evidence was identified in patients with mild preoperative kidney failure, who presented a higher mortality rate in the long term compared to patients whose preoperative kidney function was normal^[^^17^^]^.

It is believed that mild and severe preoperative kidney failure leads to a greater incidence of adverse events in the postoperative period^[^^19^^,^^20^^]^. In this study, preoperative kidney failure requiring dialysis was not related to higher mortality rates. The patients with preoperative kidney failure (GFR V) presented greater postoperative survival in the first 30 days and over time when compared to the remaining patients, which may be associated with early dialysis therapy on the post-operative period.

Patients undergoing dialysis in whom myocardial revascularization was performed present higher mortality, approximately 7% to 10%, which is three times greater than non-uremic patients^[^^20^^]^. Long-term results show that the survival of patients undergoing dialysis after coronary artery bypass graft (CABG) is decreased, with mortality in 5 years estimated to be 48%^[^^6^^,^^11^^,^^21^^]^.

Patients who developed some degree of AKI, in addition to presenting high mortality rates, also presented a high risk score, required more blood transfusions^[^^6^^,^^20^^-^^23^^]^, presented a higher incidence of neurological events, required mechanical ventilation for longer periods, and, consequently, were hospitalized for longer periods with high rates of surgical wound infection^[^^20^^]^.

Complications emerging in the preoperative phase of cardiovascular surgeries among patients with AKI requiring dialysis significantly increase mortality rates^[^^17^^,^^19^^]^. Among potential complications, pulmonary atelectasis is reported as the main complication found in 54% to 92% of patients in the postoperative period of cardiovascular surgery^[^^24^^]^. The presence of complications identified in individuals who developed postoperative AKI that requires dialysis after a cardiovascular surgery shows that these patients may require mechanical ventilation for longer periods. On the other hand, mechanical ventilation increases the risk of pulmonary infection, while AKI creates an altered inflammatory environment that may cause atrial arrhythmias^[^^6^^]^.

One recent study revealed that AKI stage 1 and stages 2 or 3, according to AKIN criteria, were associated with significant increase, from 31% to 98% of readmission in five years, in addition to significant higher mortality risk, from 1.5 to 3.5 times, five years after surgery^[^^25^^]^. Similar results were found in association with increased intra-hospital mortality among patients who initiated dialysis later on (>3 days) after cardiovascular surgery, in comparison to those who started dialysis within three days after surgery^[^^9^^]^.

In this study, 140 out of the 157 patients presented some degree of preoperative kidney disease and only 17 presented normal kidney function. All the patients, however, required postoperative dialysis. Univariate analysis revealed significant differences between the groups GFR IV (severe) and GFR V (renal failure) in the pairwise analysis, with P=0.0109, though the GFR was not significant in the survival of these patients, according to the Cox regression model. It is possible that the fact that preoperative dialysis patients were introduced earlier to dialysis than those who developed postoperative AKI and later required it explains the greater survival rate among the first group of patients. Patients in a previous dialysis program were not related to have long term mortality than new demanding dialysis patients after cardiovascular surgery.

## CONCLUSION

Data show an average survival of 546 days among dialytic AKI patients after cardiovascular surgery, while factors such as age, and postoperative complications were determinant regarding decreases in the survival of these patients.

The multidisciplinary staff in the intensive care unit must be aware of the profile of these patients and understands how their clinical condition develops to refer them to early dialysis in order to properly handle AKI and decrease irreversible damage. The planning of interventions in the perioperative period is essential to decreasing the emergence of AKI and avoiding complications, significantly contributing to decreased mortality and improved survival of patients.

**Table:** 

Authors' roles & responsibilities	
ABVS	Substantial contributions to the conception, acquisition, analysis and interpretation of data for the work; final approval of the version to be published
AMRZC	Contributions on interpretation of data, drafting the work or revision it critically for important intellectual content; final approval of the version to be published
FPT	Contribution to the conception and design of the work, final approval of the version to be published and agreement to be accountable for all aspects of the work in ensuring that questions related to the accuracy or integrity of any part of the work are appropriately investigated and resolved; final approval of the version to be published
